# Nucleus pulposus phenotypic markers to determine stem cell differentiation: fact or fiction?

**DOI:** 10.18632/oncotarget.6782

**Published:** 2015-12-28

**Authors:** Abbey A. Thorpe, Abbie L.A. Binch, Laura B. Creemers, Christopher Sammon, Christine L. Le Maitre

**Affiliations:** ^1^ Biomolecular Sciences Research Centre, Sheffield Hallam University, Sheffield, UK; ^2^ UMC Utrecht, Orthopaedics Department, Utrecht, Netherlands; ^3^ Materials and Engineering Research Institute, Sheffield Hallam University, Sheffield, UK

**Keywords:** nucleus pulposus, phenotypic markers, regeneration, stem cells PAX1, FOXF1, Pathology Section

## Abstract

Progress in mesenchymal stem cell (MSC) based therapies for nucleus pulposus (NP) regeneration are hampered by a lack of understanding and consensus of the normal NP cell phenotype. Despite the recent consensus paper on NP markers, there is still a need to further validate proposed markers. This study aimed to determine whether an NP phenotypic profile could be identified within a large population of mature NP samples.

qRT-PCR was conducted to assess mRNA expression of 13 genes within human non-degenerate articular chondrocytes (AC) (*n*=10) and NP cells extracted from patients across a spectrum of histological degeneration grades (*n*=71). qRT-PCR results were used to select NP marker candidates for protein expression analysis.

Differential expression at mRNA between AC and non-degenerate NP cells was only observed for Paired Box Protein 1 (PAX1) and Forkhead box F1 (FOXF1). In contrast no other previously suggested markers displayed differential expression between non-degenerate NP and AC at mRNA level. PAX1 and FOXF1 protein expression was significantly higher in the NP compared to annulus fibrosus (AF), cartilaginous endplate (CEP) and AC. In contrast Laminin-5 (LAM-332), Keratin-19 (KRT-19) and Hypoxia Inducible Factor 1 alpha (HIF1α) showed no differential expression in NP cells compared with AC cells.

A marker which exclusively differentiates NP cells from AF and AC cells remains to be identified, raising the question: is the NP a heterogeneous population of cells? Or does the natural biological variation during IVD development, degeneration state and even the life cycle of cells make finding one definitive marker impossible?

## INTRODUCTION

Low back pain (LBP) is one of the most prevalent health problems in the western world [1, 2], with degeneration of the intervertebral disc (IVD) implicated in 40% of cases [3, 4]. Current surgical treatments have been directed towards alleviating patient symptoms[5] but can accelerate degenerative changes in adjacent discs and have poor outcomes [6, 7]. New approaches in tissue engineering have provided a variety of potential treatment options [8]. However current progress in MSC based therapies are hampered by a lack of understanding and consensus of the normal nucleus pulposus (NP) cell phenotype [9]. Considerable progress in understanding the ontogeny and physiology of NP cells has occurred over the last decade with a resultant plethora of marker genes identified to distinguish NP from annulus fibrosus (AF) and articular chondrocytes (AC) [9-14]. Risbud *et al.,* (2015) recently reported NP phenotypic markers recommended for use in directing MSC based regeneration strategies for the NP [9]; this paper focused on defining the young healthy NP cell phenotype, as they hypothesised this cell type would be most successful in terms of NP regeneration as a treatment strategy for LBP [9]. However, despite this consensus paper, there is still a need to further validate, at gene and protein level, proposed NP markers which have been identified from transcription expression profiles or assessed at protein level but only in a small number of human samples; such molecules include: paired box protein 1 (PAX1), forkhead box f1 (FOXF1) [10, 15] and ovostatin-2 (OVO-2) [10] as well as the proposed NP negative marker Integrin binding sialoprotein (IBSP)[10], shown to be differentially expressed between NP and AC cells. Moreover the potential for matrix binding proteins to act as indicators of the NP ECM warrant further investigation. Namely laminin-332 (laminin-5) and laminin-511 (laminin 10) which have been previously shown to be expressed highly in rat and porcine NP tissue in comparison to annulus fibrosus (AF) tissue, however to date this has not been investigated in human IVD tissue [16, 17].

The aim of this study was to further validate the use of proposed NP specific markers on a large cohort of human NP and AC samples, at mRNA and protein level using qRT-PCR and immunohistochemistry. To determine whether an NP phenotypic profile could be identified within a large population of mature NP samples.

## RESULTS

### qRT-PCR analysis

**Figure 1:**
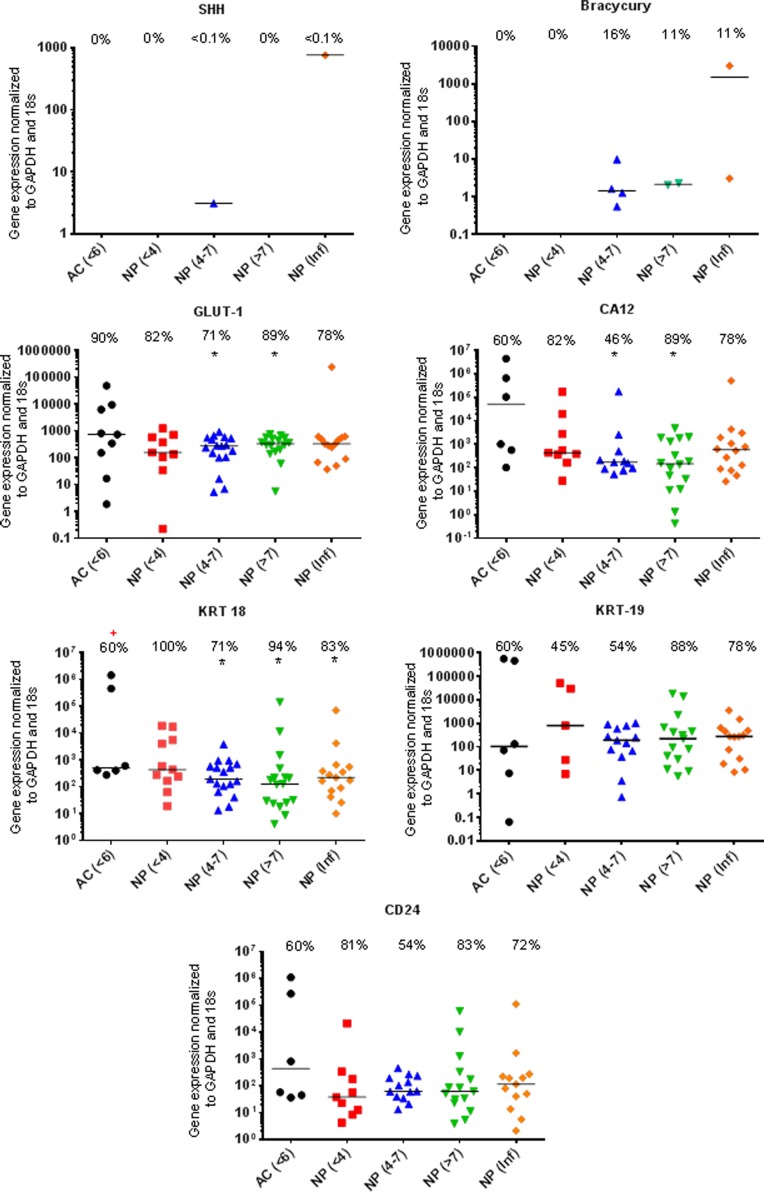
Markers recommended for use in defining the young healthy NP phenotype qRT-PCR mRNA expression from directly extracted articular chondrocytes (AC), histologically non degenerate (<6) and directly extracted nucleus pulposus (NP) cells, graded histologically as non-degenerate (<4); moderately degenerate (4-7); severely degenerate (>7) and those containing infiltrated cells (Inf). Percentage of positive samples displayed above. (*) significant difference in expression levels between AC and NP cells. (+) Significant difference in proportion of samples expressing target gene in NP samples compared to AC samples: *P* = ≤0.05.

**Figure 2:**
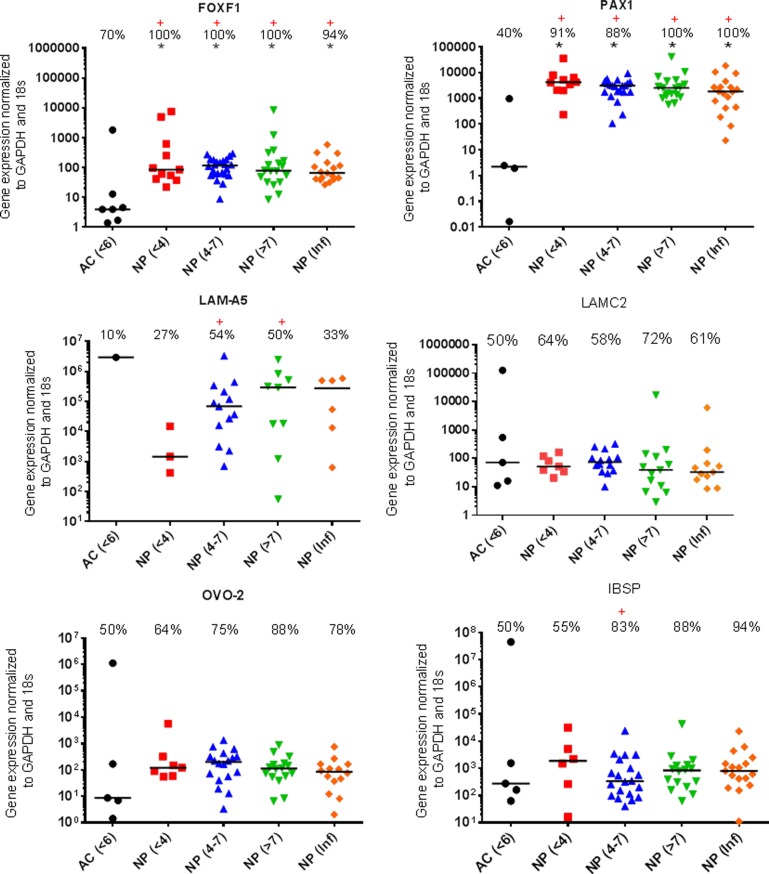
Proposed NP markers for further investigation qRT-PCR mRNA expression from directly extracted articular chondrocytes (AC), histologically non degenerate (<6) and directly extracted nucleus pulposus (NP) cells, graded histologically as non-degenerate (<4); moderately degenerate (4-7); severely degenerate (>7) and those containing infiltrated cells (Inf). Percentage of positive samples displayed above. (*) significant difference in expression levels between AC and NP cells. (+) Significant difference in proportion of samples expressing target gene in NP samples compared to AC samples: *P* = ≤0.05.

Previously identified ‘marker’ genes SHH, Brachyury, GLUT-1, CA12, KRT18, KRT19, CD24 (Figure 1), FOXF1, PAX1, LAM-511 (identified by α5) LAM-332 (identified by γ2), OVO-2 as well as IBSP (Figure 2), a proposed negative NP marker, were assessed for mRNA expression using qRT-PCR. No significant difference in the levels of mRNA expression were observed between AC compared with non-degenerate NP cells with the exception of PAX1 and FOXF1 (Figures 1 & 2).

The proportion of samples found to be expressing KRT-18 was significantly lower (P=0.0099) in AC samples compared with NP samples (Figure 1). The proportion of samples found to be expressing LAM-A5 was also significantly higher in moderate (4-7) (P=0.0086) and severely degenerate NP cells (>7) (P=0.0171) in comparison to AC cells (P=0.0317) (Figure 2). The notochordal markers SHH and brachyury were expressed in a small proportion of NP samples, with no expression detected in the non-degenerate (<4) NP sample cohort (Figure 1). The proportion of samples found to be positive for IBSP mRNA expression was significantly higher (P=0.0263) in moderately degenerate (4-7) NP samples compared with AC samples (Figure 2). PAX1 and FOXF1 showed significantly higher expression levels in NP cells in comparison to AC cells (PAX1: P=0.0003) (FOXF1: P=0.0049) accompanied by a significantly higher proportion of samples (PAX1: P=0.0024) (FOXF1: P=0.0067) in NP samples compared with AC samples, regardless of degeneration grade (Figure 2).

### Differential protein expression within the anatomical regions of the IVD

**Figure 3:**
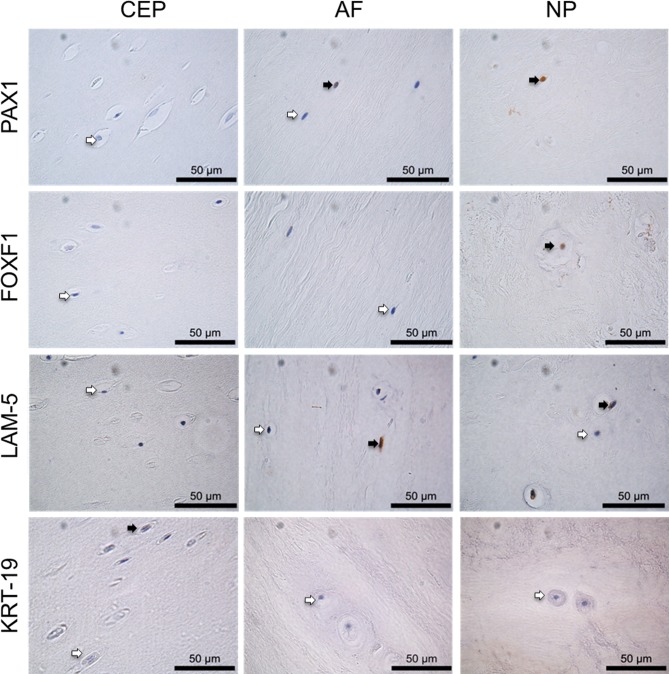
Immunohistochemistry of post mortem intervertebral disc samples IHC of PAX1, FOXF1, LAM-5 and KRT-19 on post mortem samples, CEP (cartilaginous end plate), AF (annulus fibrosus) and NP (nucleus pulposus). Positive cells indicated by black arrows, negative cells indicated by white arrows. Scale bar 50μm.

**Figure 4:**
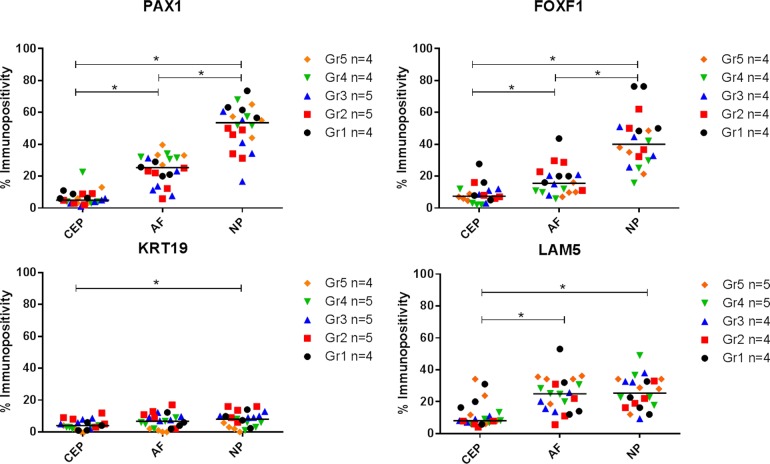
Immunopositive quantification of post mortem intervertebral disc samples Thompson grades 1-5. 200 cells counted per anatomical region and % immunopositivity calculated. (*) Significance indicated between expression levels *P* ≤ 0.05.

KRT-19 protein was expressed at low levels throughout all anatomical regions of the IVD and was significantly higher in in the NP in comparison to the CEP (P≤0.05) (Figures 3 & 4). LAM-5 (LAM-332) protein expression was detected in all regions with significantly higher expression levels in the NP (P=0.0001) and AF (P=0.0001) in comparison to the CEP, however no significant difference in the expression of LAM-5 protein was observed between the AF and NP (Figures 3 & 4). PAX1 and FOXF1 protein were expressed in all regions of the IVD and were significantly higher in the AF (PAX: P=0.0001; FOXF1: P=0.003) and NP (PAX: P=0.0001; FOXF1: P=0.0001) in comparison to the CEP and also significantly higher in the NP (PAX: P=0.0001; FOXF1: P= 0.0001) compared to the AF (Figures 3 & 4).

### Immunohistochemical validation of NP marker genes in human AC and NP samples

**Figure 5:**
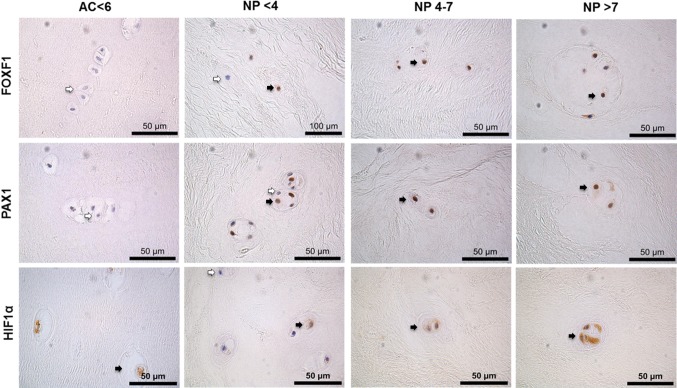
Immunohistochemistry of surgical intervertebral disc samples PAX1, FOXF1 and HIF1α on articular cartilage (AC) histologically graded as non-degenerate (<6) and nucleus pulposus (NP) tissue surgically removed following discectomy graded histologically as non-degenerate (<4); moderately degenerate (4-7) and severely degenerate (>7). Positive cells indicated by black arrows, negative cells indicated by white arrows. Scale bar 50μm.

**Figure 6:**
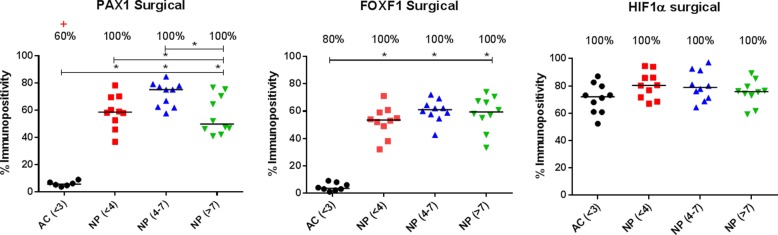
Immunopositive quantification of surgical intervertebral disc samples Immunohistochemistry of PAX1, FOXF1 and HIF1α on articular cartilage (AC) histologically non degenerate (<6) and nucleus pulposus (NP) tissue graded histologically as non-degenerate (<4); moderately degenerate (4-7) and severely degenerate (>7). Percentage of positive samples displayed above. (*) indicates significance between % immunopositivity of NP compared to AC. (+) Indicates significance between proportion of NP samples found to be expressing target protein, compared to AC samples *P* = ≤0.05.

Within surgical samples PAX1 and FOXF1 protein was expressed in 100% of NP samples and 60% of AC (PAX1) and 80% of AC (FOXF1) samples (Figures 5 & 6). The expression of PAX1 and FOXF1 was significantly higher in NP samples in comparison to AC samples regardless of grade of degeneration (P≤0.05) (Figure 6). HIF1α protein was expressed in 100% of NP and AC samples (Figure 6). No significant difference in the protein expression of HIF1α was observed between AC and NP samples (Figure 6).

## DISCUSSION

### NP markers indicative of NP ontongeny

The origin of NP cells is currently an unresolved area of IVD research. Compelling evidence indicates that mature NP cells of the adult IVD have differentiated along the notochordal lineage [30], however existing evidence which supports the migration of CEP and AF cells should not be ignored [31, 32]. The notochordal origin is reflected in the recommended markers SHH and brachyury expressed in the developing notochord [33-35]. Decreased SHH and brachyury expression have been shown to correlate with aging and degeneration which agrees with the low proportion of samples expressing SHH and brachyury in this study, which could be due to the age of patient samples enrolled in this study (20-71yrs).[36] Recently, however Wnt mediated reactivation of SHH signalling has been shown to increase expression of brachyury, aggrecan and chondroitin sulphate in aged discs [37]. Expression of brachyury has also been reported in the mature NP [11, 36]. The lack of notochordal cells within the mature healthy adult NP must be considered when developing regenerative treatment strategies for IVD degeneration, since transplanted cells need to be capable of withstanding the altered microenvironmental cues in the mature NP. An ideal phenotypic NP marker would be one which is NP specific, reflects NP ontogeny and is consistently expressed in healthy mature NP cells, which populate and survive in the adult IVD. Having said this, using markers which reflect NP ontogeny, to inform differentiation of regenerative cells into NP cells is likely to be problematic since a lack of expression of such markers does not necessarily demonstrate that the regenerative cells being used are not differentiating into NP cells, it may simply demonstrate that the regenerative cells are not from the same ontogeny as NP cells.

### NP markers indicative of NP physiology

The NP is the largest avascular tissue in the human body and thus NP cells are physiologically adapted to survive in a hypoxic microenvironment, mediated by the expression of HIF1α [27, 29, 38]. HIF-1α drives glycolytic metabolism and supports the function and survival of NP cells by inducing the up-regulation of GAPDH, GLUT1/3, galectin-3, glucuronyltransferase-1 and vascular endothelial growth factor-A (VEGF-A) [28, 29]. GLUT-1 in particular, has been included alongside HIF1α as a recommended marker, reflective of NP physiology [9, 14]. However no differential expression was observed between AC and NP cells for GLUT1 or HIF1α. Similarly CA12, a physiological marker which reflects the ability of NP cells to buffer the elevated lactic acid found in this tissue, [39] was also expressed in AC and NP cells with no significant difference in expression levels. Akin to the NP, AC is an avascular, hypoxic environment [40], and HIF-1α is crucial for AC cell survival [41]. Thus, although physiological markers are essential to assess the ability of differentiated cells to respond appropriately to the harsh NP microenvironment, they are not NP specific. Interestingly however NP cells uniquely constitutively express HIF1α even in the presence of oxygen [27]. NP cell differentiation assessment could focus on constitutive HIF1α expression under hypoxic and normoxic culture conditions as opposed to simply expression of the protein.

### Differentially expressed markers recommended for use in defining the NP phenotype

Microarray studies by Fujitta *et al.,* 2005 identified the expression of the heat-stable antigen CD24, in rat NP cells, which was later replicated in subsequent studies [42, 43]. Despite this, differential expression of CD24 was not confirmed in human samples in this study, in agreement with Rutges *et al.,* 2010 who reported that CD24 was not differentially expressed [12]. KRT-18 (canine) and KRT-19 (rat) were also identified as differentially expressed between NP and AC cells [25], which was confirmed in bovine IVDs [11]. However, these results failed to translate to human samples in this study with no significant difference observed. KRT-19 is expressed in the embryonic notochord [44] and has been shown to decrease with age [12] which could explain the differential results in this study. Although KRT-18 was seen in human NP samples 67% of AC samples also showed expression, thus the use of this marker alone lacks NP specificity.

### Differentially expressed markers between NP and AC which require further investigation

A multitude of proposed genes have been identified as NP phenotypic markers, those which are less well validated include the OVO-2 [10], FOXF1 [10, 15] and PAX1 [10, 15]. Additionally LAM-332 (LAM-5) and LAM-511 (LAM-10) have recently been shown to be differentially expressed between porcine and rat NP and AF [17]. Furthermore it may also be useful to include NP negative, AC positive markers in a panel which assesses the phenotypic profile of differentiating cells to NP-like cells, such as IBSP [10]. Unlike previous studies OVO2, LAM-511 (identified by the α5 subunit), LAM-332 (identified by the _γ_2 chain), and IBSP were not differentially expressed at mRNA level between NP and AC in human samples. The higher protein expression of LAM-5 in NP compared to AF reported previously [16] was not replicated in human samples in this study, however the LAM-5 antibody used recognises the α3 subunit and thus may also recognise LAM-6/7. A variety of studies have published conflicting data on marker genes proposed as being differentially expressed between NP and AC; this could be due to species variation and/or small sample sizes.

Previously reported novel NP marker genes, PAX1 and FOXF1 were validated by qRT-PCR and immunohistochemistry as being more highly expressed in the NP than AC [10, 15]. Interestingly, protein expression of PAX1 and FOXF1 were also higher in NP than both AF and the CEP, shown here for the first time in native tissue; supported recently by Van den Akker *et al.,* 2014, where human NP and AF cell populations were extracted from non-degenerate healthy IVDs and were shown to express PAX1 and FOXF1 more highly in extracted NP compared to AF cells [15]. Higher expression of FOXF1 in NP cells compared to AC cells has been previously reported [10, 15], but the exact role of FOXF1 in the human IVD is yet to be elucidated. The role of FOXF1 in cell proliferation, differentiation and cell survival is well established [45-47]. FOXF1 gene deletions have been associated with birth defects including spinal malformations and fusion of the vertebrae [48]. PAX1 expression in the IVD has been previously reported in young adult mice [49], in the human fetal vertebral column [50] and in the human NP [10], however, the exact role of PAX1 within the NP is unknown.

Both PAX1 and FOXF1 can be activated by SHH signalling. [51] PAX1 is regulated by SHH signalling and is often used to illustrate continued SHH signalling in sclerotome development [52]. While SHH is not necessary for the induction of PAX1, SHH knockout mice lack vertebral structures and quickly lose PAX1 expression [53], thus, the loss of SHH expression in mature adult NP cells could alter the expression of PAX1 in human NP cells. Although PAX1 expression has previously been associated as a marker of sclerotomal cells and is highly expressed in the AF, shown in mouse models [49, 54], studies which assess the expression of PAX1 and FOXF1 during human IVD development and in the different anatomical regions of the IVD are lacking. Whether the high expression of PAX1 and FOXF1 shown here in mature human NP cells reflects NP cell ontogeny, possible migration of cells from adjacent tissues, or is a functional marker of mature adult NP cells, remains to be elucidated. Despite limited understanding of the role of PAX1 and FOXF1 in the human NP, this study demonstrated differential expression by NP cells which did not increase with degeneration grade; thus the exact role of PAX1 and FOXF1 within the NP warrants further investigation and both should be considered for use in defining the adult NP cell phenotype. Noteworthy, however is the fact that although expression levels were higher in NP cells, AC and AF cells were shown to express PAX1 and FOXF1; thus it is vital that such molecules are only used within a panel of NP markers thus providing a phenotypic profile which reflects the ontogeny, physiology and function of NP cells.

## CONCLUSIONS

A marker which exclusively differentiates NP cells from AF and AC cells remains to be identified, raising the question: is the NP a heterogeneous population of cells? Or does the natural biological variation during IVD development, degeneration state and even the life cycle of cells make finding one definitive marker impossible? From a clinical perspective the aim is to restore/maintain spine biomechanics and alleviate patient symptoms; thus the ability of differentiated cells to recapitulate the NP ECM (aggrecan to collagen type II ratio >20) and NP biomechanics should be the prime focus when developing treatment strategies for regeneration of the IVD, as opposed to the expression of NP phenotypic markers.

## MATERIALS AND METHODS

### Human IVD tissue

Human IVD tissue was obtained from surgery or PM examination (processed within 72 hours after death) with informed consent of the patients or relatives. Ethical approval was obtained from Sheffield Research Ethics Committee (09/H1308/70), and department of Pathology/UMCU Biobank, UMC Utrecht (Supplementary Table I/II).

### Human articular cartilage tissue

Human articular cartilage (AC) samples were obtained from patients undergoing total knee replacement surgery with informed consent of the patient or relatives (Supplementary Table III). Cartilage was obtained under the National Research Ethics Service approval held by the Sheffield Musculoskeletal Biobank. AC was obtained from various anatomical compartments within the knee (medial and lateral tibio-femoral and patello-femoral compartments) for 10 surgical AC samples. Cartilage tissue was graded macroscopically 0–4 using the Outerbridge classification [18]. All AC samples used in this study were macroscopically graded as non-degenerate (grade 0-1).

### Tissue processing

#### IVD tissue

IVD tissues processed at Sheffield Hallam University (SHU) were separated into two and either fixed in 10% neutral buffered formalin (Leica, Milton Keynes UK) and processed to paraffin wax (Leica) or processed for cell extraction. Following paraffin wax embedding, 4μm sections were cut, mounted onto positively charged slides (Leica) and IVD tissue histologically graded using previously published criteria [19, 20]. NP tissue free from contaminating AF and CEP was digested in protease and collagenase to isolate cells and RNA extracted as previously published [21]. IVD Tissue processed at Utrecht was decalcified and embedded to paraffin wax as previously published [22]. Four micron sections were histologically graded using previously published criteria [19, 20].

#### AC tissue

AC tissue was separated into two and either fixed in 10% v/v neutral buffered formalin (Leica) and processed to paraffin wax, or processed for cell extraction. Following paraffin wax embedding, 4μm sections were histologically graded based on the Mankin [23] grading system with the addition of abnormal features and cartilage thickness also assessed. Cells were extracted using trypsin and collagenase as previously published [24], and cells were used for direct RNA extraction using TRIZOL reagent and cDNA synthesised.

### Real-time quantitative polymerise chain reaction (qRT-PCR)

Target genes were investigated using quantitative real-time polymerase chain reaction (qRT-PCR) conducted on 10 non-degenerate AC samples (microscopic grade 0-6), 11 non-degenerate NP samples (grade <4), 24 moderately degenerate NP samples (grade 4-7), 18 severely degenerate NP samples (grade >7) and 18 NP samples with evident infiltration (Supplementary Table I, II, III).

qRT-PCR was performed on a StepOnePlus™ Real-Time PCR System (Applied Biosystems, Lutterworth UK) for potential NP markers (Pre-designed primer/probe mixes Applied Biosystems) (Supplementary Table IV). Glyceraldehyde-3-phosphate dehydrogenase (GAPDH) and 18S (Applied Biosystems) were used as housekeeping genes. Ten microliter reactions were prepared using TaqMan Universal PCR Master Mix (Applied Biosystems). Results were analysed using the 2^−ΔCt^ method and presented as relative gene expression normalised to the average C_T_ for the two housekeeping genes.

### Immunohistochemistry

Immunohistochemistry (IHC) was conducted on PM samples from the department of Pathology/UMCU Biobank, UMC Utrecht, Thompson grades 1-5 (n=4/5) to assess protein expression within each of the anatomical regions (CEP, AF, NP) of the IVD. IHC analysis was undertaken for PAX1 and FOXF1, found to be differentially expressed at gene level (Figure 2), KRT-19 due to its extensive use as an NP marker in a variety of studies [10, 12, 25] and LAM-5 (LAM-332) to determine whether the differential expression observed between NP and AF in porcine tissue correlated with human tissue [16, 17]. Further IHC analysis of PAX1 and FOXF1, shown to be differentially expressed within the NP compared to both the AF and CEP (Figures 3 & 4) and HIF1α shown to be essential to the physiology of NP cells and regulated at protein level [26-29], was conducted on a larger subset of surgical samples (NP: n=30; AC: n=10) (Supplementary Table I/III) to investigate differential expression between NP and AC tissue.

Sections were prepared as described for histological analysis; IHC was performed as previously described, [19] specific antigen retrieval, serum block, and antibody details shown in Supplementary Table V. All slides were visualised using an Olympus BX51 microscope and images captured by digital camera and Capture Pro OEM v8.0 software (Media Cybernetics, Buckinghamshire, UK). Evaluation of IHC staining was performed by counting 200 cells within each anatomical region (NP, AF, CEP or AC) on each section, with immunopositive cells expressed as a percentage of total count.

### Statistical analysis

Data were shown to be non-parametric and hence a Kruskall-Wallis with Conover-Inman post hoc analysis test was used to identify significant differences between AC compared with NP samples, as well as differences between NP degeneration grade, investigated by qRT-PCR and IHC *(P≤0.05)*. Two independent proportionality tests were performed to identify differences between the proportions of samples expressing the target molecules at mRNA and protein level *(P≤0.05)*.

## SUPPLEMENTARY MATERIAL TABLES



## Abbreviations

MSC: mesenchymal stem cell
LBP: low back pain
NP: nucleus pulposus
AF: annulus Fibrosus
CEP: cartilagenous endplate
AC: articular chondrocyte
SHH: sonic hedgehog
GLUT-1: glucose transporter 1
CA12: carbonic anhydrase XII
KRT18: Keratin 18
KRT19: Keratin 19
CD24: Cluster of differentiation 24
FOXF1: Forkhead Box F1
PAX1: Paired Box 1
LAMA5: Laminin Alpha 5
LAMC2: Laminin gamma 2
LAM5/332: Laminin 5/332
LAM10/511: Laminin, 10/511
OVO-2: Ovostatin 2
IBSP: Integrin Binding Sialoprotein
HIF1α: Hypoxia-inducible factor 1-alpha
