# Supplementary material for: Nucleus pulposus phenotypic markers to determine stem cell differentiation: fact or fiction?
Source: Oncotarget. 2015 Dec 28;7(3):2189–200. doi: 10.18632/oncotarget.6782 (PMC4823028; doi:10.18632/oncotarget.6782)
Supplement: Supplementary file 1 [file oncotarget-07-2189-s001.pdf]

Nucleus pulposus phenotypic markers to determine stem cell differentiation: fact or fiction?

Supplementary Material

Supplementary Table I: IVD samples processed at Sheffield Hallam University: Patient Details.

Reference		Source	IVD Level	Infiltrating Cells	Average Grade	Classification	DE	IHC
**HD**	1	Surgical	L4/L5	No	3.0	I	✓	✓
**HD**	2	Surgical	L5/S1	Yes	7.0	I	✓	
**HD**	3	Surgical	L4/L5	No	4.0	MD	✓	
**HD**	5	Surgical	L5/S1	No	8.5	SD	✓	
**HD**	9	Surgical	L5/S1	No	2.0	ND		✓
**HD**	17	Surgical	L5/S1	Yes	4.0	ND	✓	
**HD**	21	Surgical	L4/L5	Yes	4.0	I	✓	
**HD**	24	Surgical	L5/S1	No	2.0			✓
**HD**	25	Surgical	L4/L5	No	2.0	I	✓	
**HD**	26	Surgical	L5/S1	No	5.0	I	✓	
**HD**	30	PM (SHU)	L4/L5	No	2.0		✓	
**HD**	31	PM (SHU)	L3/L4	No	3.0	ND		✓
**HD**	34	Surgical	L5/S1	Yes	12.0	SD	✓	
**HD**	44	Surgical	L5/S1	No	2.0	ND	✓	
**HD**	45	Surgical	L5/S1	No	8.0	SD	✓	
**HD**	53	Surgical	L5/S1	No	7.0	MD	✓	
**HD**	54	Surgical	L4/L5	No	6.0	SD	✓	✓
**HD**	56	Surgical	L5/S1	Yes	5.0	MD	✓	✓
**HD**	57	Surgical	L5/S1	No	6.0	MD	✓	✓
**HD**	58	Surgical	L5/S1	No	3.5	SD	✓	
**HD**	59	Surgical	L5/S1	No	6.0	SD	✓	
**HD**	61	Surgical	L5/S1	No	7.5	MD	✓	✓
**HD**	63	Surgical	L5/S1	No	9.0	SD	✓	✓
**HD**	65	Surgical	L4/L5	No	10.0	SD	✓	
**HD**	66	Surgical	L3/L4	Yes	5.0	I	✓	
**HD**	71	Surgical	L5/S1	Yes	3	MD		✓
**HD**	75	Surgical	L3/L4	No	8.0	MD	✓	✓
**HD**	79	Surgical	L4/L5	No	3.0	ND	✓	✓
**HD**	85	Surgical	L2/L3	No	8.0	I	✓	
**HD**	86	Surgical	L5/S1	Yes	1.0	I	✓	
**HD**	92	Surgical	L5/S1	No	3.5	ND	✓	
**HD**	93	Surgical	L5/S1	Yes	6.0	MD	✓	✓
**HD**	94	Surgical	L5/S1	No	6.0	MD	✓	
**HD**	97	Surgical	L5/S1	No	6.0	MD	✓	✓

**HD**	98	Surgical	L3/L4	No	6.0	MD	✓	
**HD**	109	Surgical	L4/L5		6.0			
**HD**	133	Surgical	L4/L5	No	3.5			✓
**HD**	144	Surgical	L5/S1	No	5.0		✓	✓
**HD**	145	Surgical	L4/L5	No	11	SD	✓	
**HD**	146	Surgical	L5/S1	No	5.85	MD	✓	
**HD**	148	Surgical	C5/C6	No	6.0	MD	✓	
**HD**	151	Surgical	L5/S1	No	5.3	MD	✓	
**HD**	153	Surgical	L5/S1	Yes	5.0	MD	✓	
**HD**	154	Surgical	L4/L5	No	3.0	MD	✓	
**HD**	156	Surgical	L4/L5	Yes	3.0	ND	✓	
**HD**	157	Surgical	L5/S1	Yes	5.0	I	✓	
**HD**	158	Surgical	L4/L5	No	3.0	I	✓	✓
**HD**	159	Surgical	L4/L5	Yes	9.0	I	✓	
**HD**	160	Surgical	C3/C5	No	6.0	MD	✓	
**HD**	166	Surgical	L4/L5	No	8.0	SD	✓	
**HD**	170	Surgical	L5/S1	No	8.0	SD		✓
**HD**	174	Surgical	L5/S1	No	7.0	MD	✓	
**HD**	175	Surgical	L5/S1	No	7.0	SD	✓	
**HD**	177	Surgical	L4/L5	No	2.0	N	✓	
**HD**	184	Surgical	L4/L5	Yes	5.0	SD	✓	
**HD**	192	Surgical	L5/S1	No	8.0	SD	✓	
**HD**	194	Surgical	L4/L5	No	11.0	SD	✓	✓
**HD**	195	Surgical	C6/C7	No	9.0	SD	✓	
**HD**	197	Surgical	L4/L5	No	8.0	SD	✓	
**HD**	203	Surgical	S1	No	3.5			✓
**HD**	207	Surgical	L5/S1	No	8.0	MD	✓	✓
**HD**	219	Surgical	L5/S1	No	10.5	I	✓	
**HD**	225	Surgical	L5/S1	No	10.0	SD	✓	✓
**HD**	228	Surgical		No	6.0	I	✓	
**HD**	229	Surgical	L5/S1	No	6.0	MD	✓	✓
**HD**	231	Surgical	L4/L5	No	5.0	MD	✓	✓
**HD**	232	Surgical	L5/S1	No	8.0	SD	✓	✓
**HD**	233	Surgical	L5/S1	No	9.5	SD	✓	✓
**HD**	234	Surgical	L5/S1	No	8.5	SD	✓	✓
**HD**	243	Surgical	L4/L5	Yes	6.0	I	✓	
**HD**	246	Surgical	L5/S1	Yes	6.0	I	✓	
**HD**	253	Surgical	C5/C6	Yes	4.0	I	✓	
**HD**	254	Surgical	L4/L5	Yes	6.0	I	✓	✓
**HD**	257	Surgical	L5/S1	Yes	6.0	I	✓	
**HD**	266	Surgical	L5/S1	No	4.0		✓	✓
**HD**	276	Surgical	C5/C6	No	6.0	MD	✓	✓
**HD**	281	Surgical	L4/L5	No	3.0	ND	✓	

Supplementary Table II: IVD samples processed at Utrecht: Patient Details

Reference		Source	IVD Level	Thompson Grade	DE	IHC
**HD**	S06 115	PM (UT)	L4/5	1		✓
**HD**	S08 263	PM (UT)	L4/5	1		✓
**HD**	S06 69	PM (UT)	L4/5	1		✓
**HD**	S06 123	PM (UT)	L4/5	1		✓
**HD**	S07 099	PM (UT)	L4/5	2		✓
**HD**	S07184	PM (UT)	L4/5	2		✓
**HD**	S06116	PM (UT)	L4/5	2		✓
**HD**	S06224	PM (UT)	L4/5	2		✓
**HD**	S07 099	PM (UT)	L4/5	2		✓
**HD**	S07203	PM (UT)	L4/5	3		✓
**HD**	S07229	PM (UT)	L4/5	3		✓
**HD**	S06127	PM (UT)	L4/5	3		✓
**HD**	S07098	PM (UT)	L4/5	3		✓
**HD**	S07137	PM (UT)	L4/5	3		✓
**HD**	S07209	PM (UT)	L4/5	4		✓
**HD**	S07250	PM (UT)	L4/5	4		✓
**HD**	W07243	PM (UT)	L4/5	4		✓
**HD**	S06141	PM (UT)	L4/5	4		✓
**HD**	S07256	PM (UT)	L4/5	4		✓
**HD**	S06150	PM (UT)	L4/5	5		✓
**HD**	S06251	PM (UT)	L4/5	5		✓
**HD**	S06287	PM (UT)	L4/5	5		✓
**HD**	S0741	PM (UT)	L4/5	5		✓
**HD**	S06165	PM (UT)	L4/5	5		✓

Supplementary Table III: Articular Cartilage samples: Patient Details.

Reference		Source	Anatomical Compartment	Macroscopic Grade	Microscopic Grade	DE	IHC
**HC**	4 (3)	Surgical	Medial femoral condyle	1	4	✓	✓
**HC**	5 (2)	Surgical	Posterior femoral condyle	0	5	✓	✓
**HC**	7 (1)	Surgical	Posterior femoral condyle	0	3.5	✓	✓
**HC**	9 (1)	Surgical	Posterior femoral condyle	0	2.5	✓	✓
**HC**	10 (1)	Surgical	Trochlear	0	5	✓	✓
**HC**	11 (1)	Surgical	Posterior condyle	0	3.5	✓	✓
**HC**	16 (1)	Surgical	Posterior femoral condyle	1	2.5	✓	✓
**HC**	17 (1)	Surgical	Medial femoral condyle	0	3	✓	✓
**HC**	18 (1)	Surgical	Laterial posterior femoral condyle	1	3	✓	✓
**HC**	19 (1)	Surgical	Lateral femoral condyle	0	5	✓	✓

Table IV: Predesigned primer probes for qRT-PCR

Gene	Gene Symbol	Catalogue no.
Glyceraldehyde-3-phosphate dehydrogenase	GAPDH	Hs02758991_g1
18S Ribosomal RNA	18S	Hs03003631_g1
Forkhead Box F1	FOXF1	Hs00230962_m1
Paired Box 1	PAX1	Hs01071292_m1
Keratin 19	KRT19	Hs01051611_gH
Keratin 18	KRT18	Hs02827483_g1
Cluster of differentiation 24	CD24	Hs03044178_g1
Glucose transporter 1	SLC2A1	Hs00892681_m1
Brachyury	T	Hs00610080_m1
carbonic anhydrase XII	CA12	Hs01080909_m1
Sonic Hedgehog	SHH	Hs00179843_m1
Laminin Alpha 5	LAMA5	Hs00966585_m1
Laminin gamma 2	LAMC2	Hs01043711_m1
Ovostatin 2	OVO2	Hs00416380_m1
Integrin Binding Sialoprotein	IBSP	Hs00173720_m1

SupplementaryTable V: Target antibodies used for IHC

Target Antibody	Clonality	Optimal Dilution	Antigen Retrieval Method	Secondary Antibody	Serum Block
FOXF1 (ab23194)	Rabbit Polyclonal	1:100	Heat	Goat anti-rabbit	Goat
PAX1 (ab203065)	Rabbit Polyclonal	1:400	Enzyme	Goat anti-rabbit	Goat
KRT-19 (ab7754)	Mouse monoclonal	1:400	None	Rabbit anti-mouse	Rabbit
LAM-5 (ab78286)	Mouse monoclonal	1:800	Enzyme	Rabbit anti-mouse	Rabbit
HIF1α (ab16066)	Mouse monoclonal	1:100	None	Rabbit anti-mouse	Rabbit

Supplementary Table V: *Target antibodies used for IHC, their optimal concentrations and antigen retrieval methods. Heat antigen retrieval consisted of 10 minute microwave irradiation in 0.05M Tris buffer, pH 9.5 pre-heated to 60˚C. Enzyme antigen retrieval consisted of 30-minute incubation in TBS; 20 mM tris, 150 mM sodium chloride, 46.8mM calcium chloride dihydrate pH 7.5, containing 0.01% w/v α-chymotrypsin from bovine pancreas at 37°C.*
